# Silent Spontaneous Uterine Rupture at 36 Weeks of Gestation

**DOI:** 10.1155/2015/596826

**Published:** 2015-08-19

**Authors:** J. Y. Woo, L. Tate, S. Roth, A. C. Eke

**Affiliations:** ^1^Department of Obstetrics and Gynecology, Michigan State University/Sparrow Hospital, Lansing, MI 48912, USA; ^2^Division of Maternal Fetal Medicine, Department of Obstetrics and Gynecology, Michigan State University/Sparrow Hospital, Lansing, MI 48912, USA

## Abstract

*Introduction.* Silent spontaneous rupture of the uterus before term, with extrusion of an intact amniotic sac and delivery of a healthy neonate, with no maternal or neonatal morbidity or mortality is very rare. Very few cases have been reported in literature. *Case Presentation.* We report a case of silent spontaneous uterine rupture, found during a scheduled repeat cesarean section at 36 weeks of gestation. Patient had history of two prior classical cesarean sections. She underwent cesarean section, with delivery of a healthy male infant. She had a good postoperative recovery and was discharged on postoperative day 3. *Conclusion.* Silent spontaneous rupture of the uterus before term with extrusion of an intact amniotic sac is rare. A high index of suspicion and good imaging during pregnancy are important in making this diagnosis.

## 1. Introduction

Spontaneous uterine rupture is an uncommon but potentially life-threatening obstetrical emergency for both mother and fetus. It occurs mostly during labor in the context of a previous uterine scar. Generally, uterine rupture refers to a complete separation of all uterine layers, including the uterine serosa, and this usually occurs most commonly in the setting of classical cesarean section [1]. Classical cesarean delivery entails a vertical incision involving the upper contractile portion of the uterus. In contemporary medicine, this type of incision is often reserved for preterm breech delivery or when lower uterine incision is deemed unfeasible or unsafe [2]. The reported frequency of classical cesarean delivery is 0.3% by the Eunice Kennedy Shriver National Institute of Child Health and Human Development Maternal Fetal Medicine Unit Network (NICHD MFMU) involving 320,000 births over a 4-year period [3]. The incidence of uterine rupture varies depending on the type and location of the prior uterine incision. The American College of Obstetricians & Gynecologists (ACOG) Practice Bulletin reports a uterine rupture risk of 0.5 to 0.9 percent for women with prior cesarean undergoing trial of labor [4]. However, the overall rate for uterine rupture with previous classical cesarean ranges from 0.6 to 12 percent as cited in the literature [4–8].

Severe maternal complications secondary to uterine rupture include hemorrhage, blood transfusion, and hysterectomy. The most severe complication of uterine rupture is maternal death; even though rare, it occurs in approximately 1 in 500 uterine ruptures [9]. While asymptomatic uterine dehiscence rarely results in adverse fetal outcome, complete uterine rupture with extrusion of placenta or the fetus can be catastrophic. The risk of perinatal death after uterine rupture was found to be 8.7% in a population-based cohort study in Netherlands [10], with perinatal mortality reported as ranging from 74% to 92% in less developed countries [11]. Silent uterine rupture can be very difficult to diagnose, as the clinical features of uterine rupture, including abdominal pain, vaginal bleeding, maternal hypovolemic shock, or hemorrhage, may be absent. Multiple studies have tried to develop prediction models for uterine rupture, including sonographic evaluation of uterine scar, but none has proven to be reliable especially for previous classical cesarean sections [1, 12].

## 2. Case Presentation

We report a case of spontaneous uterine rupture, found during a scheduled repeat classical cesarean section at 36 weeks of gestation with delivery of a healthy male infant. The patient had history of two prior classical cesarean sections in 2009 and 2011. She also had multiple renal and uterine reconstructive surgeries and revisions in the years preceding her pregnancies due to congenital anomalies, including a duplicated left ureter. Her pregnancy was complicated by pyelonephritis and hydronephrosis with nephrostomy tube placement and multiple episodes of urinary tract infections that were treated. Antenatal testing including multiple biophysical profiles was done prior to delivery for difficulty in monitoring fetus with nonstress tests; no abnormal findings were observed. No fetal heart rate abnormalities were seen immediate before delivery. Patient had one episode of abdominal discomfort two days prior to scheduled delivery date, which resolved after taking one dose of Norco. She did not experience any uterine contractions prior to delivery.

**Figure 1:**
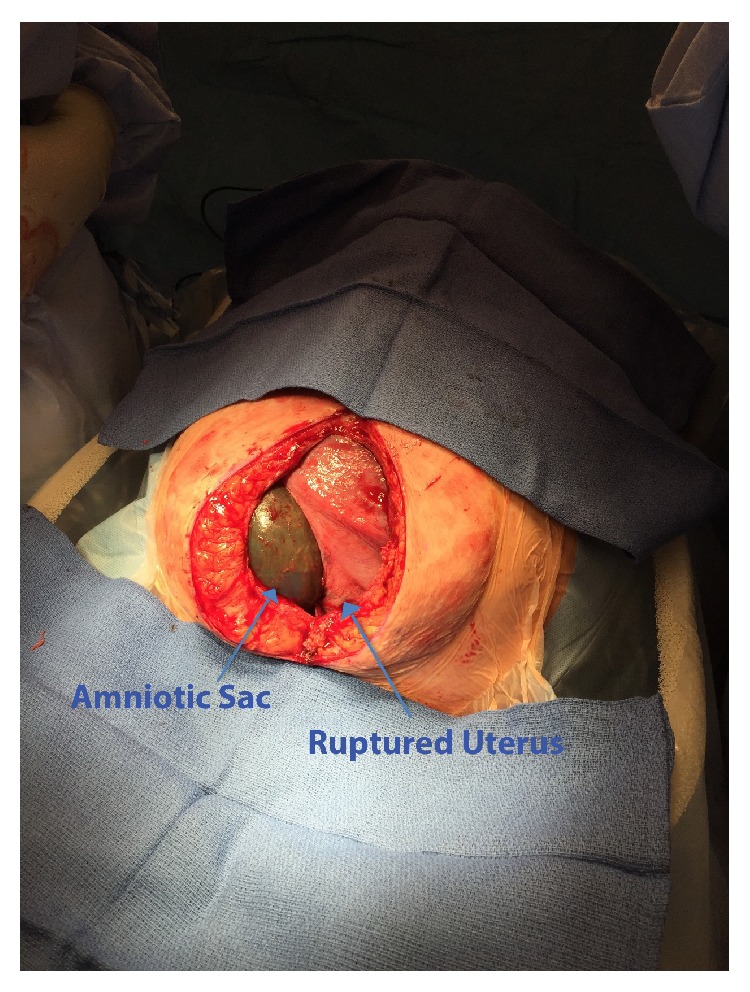
Silent uterine rupture, with extruded amniotic sac.

Upon entering the abdominal cavity via a vertical skin incision, a complete uterine rupture was seen at the prior classical incisional scar with the amniotic sac protruding into the abdomen (Figure 1). Fetal parts were palpable through the protruding membrane. No active bleeding was noted, and the uterine scar appeared to be fibrotic at both edges. The fetus was found in oblique presentation and was delivered after amniotomy in normal fashion. Neonate's APGAR at 1 and 5 minutes were 8 and 9, respectively. Birthweight was 3645 g. Inspection of the uterus revealed that the uterine scar rupture occurred left of midline due to a severely rightward rotated uterus. The posterior lower uterine segment was very thin and was ballooning outwards. Dense adhesion was noted between the bladder and anterior lower uterine segment. Uterus was repaired with multilayer closure. Tubal ligation was performed as planned. Remainder of the surgery was completed in the usual fashion.

Patient's recovery course was uncomplicated. She had normal amount of vaginal bleeding postpartum. Postoperative hemoglobin was 11.2 g/dL. Patient and newborn were discharged home in good condition on postoperative day 3.

## 3. Discussion

Uterine rupture is a serious complication of pregnancy and can cause significant maternal and perinatal morbidity, with most cases occurring in the setting of classical cesarean section. Trial of labor after cesarean (TOLAC) has been associated with higher incidence of uterine rupture [13]; however, in the case presented, patient had no signs of labor prior to delivery. She was asymptomatic, denying vaginal bleeding and abdominal pains prior to delivery. She experienced minimal abdominal discomfort (not pain) 2 days prior to delivery, which, in hindsight, may be when she ruptured her uterus. Clinical features of uterine rupture may include abdominal pain, vaginal bleeding, maternal hypovolemic shock, or hemorrhage. From our case, we learned that uterine rupture may occur without any precipitating signs or symptoms. Our patient's history of multiple pelvic surgeries can also cause unspecific and unclear symptoms.

It can be very difficult to predict individuals who would rupture their uteruses in pregnancy. Recent studies have attempted to develop predictive models for uterine rupture. Bujold [14] and colleagues developed 2 such indexes using antepartum and intrapartum factors. However, both models were neither sensitive nor specific enough for clinical use (sensitivity of 75% with false positive rate of 40%). Grobman and colleagues also developed a model to estimate specific risk of uterine rupture during trial of labor. However, the empiric probability risk of rupture derived from a wide 95% CI ranging from 0.6 to 1.8%, making this model neither accurate nor discriminating [15].

Ultrasonography has been studied to predict uterine rupture. Bujold and colleagues [14] conducted a prospective cohort study of 125 women with previous cesarean undergoing trial of labor. Their analysis determined that optimal cutoff is a lower uterine thickness of <2.3 mm, with the rate of uterine rupture being 9.1% for this group. The limitation of this study includes the fact that most women with a lower uterine thickness <2.0 mm did not undergo trial of labor. This might suggest an established practice pattern which might limit future studies using ultrasound to predict uterine rupture. For our case, the patient had multiple ultrasound studies done for growth and biophysical profiles. However, none were specifically looking for the lower uterine segment. Due to her history of classical incision, measuring the lower uterine segment might not have been adequate to evaluate uterine thickness anyway. Review of all ultrasound images in our patient revealed no abnormality.

Despite previously quoted high rate of perinatal mortality, studies done in the United States revealed much lower perinatal death rate of 0.3 per 1000 trials of labors [2]. The lower rate of perinatal death might be due to rapid recognition of and response to potential uterine ruptures.

## 4. Conclusion

This case report emphasizes that uterine rupture can occur without symptoms in pregnancy. A high index of suspicion and proper imaging are therefore needed in making this diagnosis.
